# Thermal and Rheological Properties of Hydrophobic Nanosilica in Sunflower Oil Suspensions at High Pressures

**DOI:** 10.3390/nano11113037

**Published:** 2021-11-12

**Authors:** María J. Martín-Alfonso, Javier Pozo, Clara Delgado-Sánchez, Francisco José Martínez-Boza

**Affiliations:** Centro de Investigación en Tecnología de Procesos y Productos Químicos (Pro2Tecs), Escuela Técnica Superior de Ingeniería, Universidad de Huelva, Avda. Tres de Marzo, 21071 Huelva, Spain; javier.pozo@diq.uhu.es (J.P.); clara.delgado@diq.uhu.es (C.D.-S.); martinez@uhu.es (F.J.M.-B.)

**Keywords:** sunflower oil, hydrophobic fumed silica, high-pressure, drilling fluid

## Abstract

Nowadays, the reduction of the environmental impact associated with the operation of the oil industry is a primary concern. A growing trend is to develop low-toxicity formulations based on biodegradable components. In this sense, vegetable oils structured with nanomaterials could be an alternative to mineral or synthetic oils for sustainable fluid formulations. Hydrophobic fumed silica nanoparticles have the capability to change the rheological behavior of oil in suspensions, providing a large variety of non-Newtonian behaviors over a wide range of temperatures, from shear-thinning to gel-like, depending on the concentration and the nanosilica’s hydrophobicity, that permits the design of fluids with selected characteristic and applications. This work explores the microstructure and the rheological behavior of hydrophobic fumed silica dispersed in a sunflower oil as a function of temperature and pressure. The results suggest that the suspensions of hydrophobic silica in sunflower oil reveals appropriate rheological and thermal properties over a wide range of temperatures and pressures to serve as components of sustainable drilling fluids.

## 1. Introduction

Nowadays, the reduction of the environmental impact associated with drilling operation in both geothermal and oil wells is a primary concern. Therefore, there is a current trend to search for sustainable and environmentally-friendly fluids that maintain high efficiency at minimum costs in high pressure and temperature environments (HPHT) [1]. Drilling fluids perform several tasks such as cooling and lubricating the drill bit, cleaning the hole, and protecting from formation damage. The basic formulation shows pseudoplastic behavior, consisting of a suspension of additives and rheology controllers in a continuous phase [2]. Drilling fluids are classified as water-based mud (WBM) and oil-based mud (OBM) according to the nature of its continuous phase. Despite WBM having economic and environmental advantages over OBM [3,4,5,6,7], in challenging wells OBM present technical advantages thanks to intrinsic oil properties such as high thermal stability, lubricity, and shale inhibition [8,9]. In the context of environmental concerns, fluids having a low-toxicity continuous phase such as vegetable oils and derivatives are more suitable [10].

The oil phase can have a mineral, synthetic, or vegetable origin. Oil phase behaves similarly to Newtonian liquid at all temperature ranges at which the fluid is subjected when in-service. The rheology controllers, that are frequently organoclay particles [11,12,13,14], provide the fluid with the desirable shear-thinning properties necessary to maintain cutting in suspension when the flow ceases and, at the same time, low viscosity at a high shear rate, which facilitates pumping circulation and hole cleaning [15].

Synthetic and mineral oils have been used extensively in OBM due to their good lubricating properties and thermal resistance to aggressive environments. However, they have poor biodegradability. Vegetable oils and derivatives could replace them to develop environmentally-friendly muds [16]. In this sense, some studies have explored vegetable oil formulations based on soybean oil [17,18] and palm methyl ester [19]. These fluids exhibited Bingham plastic flow behavior and fluid-loss properties comparable with those of conventional muds. Recently, Martin-Alfonso et al. [20] demonstrated that both sunflower and rapeseed oils structured with organosepiolites showed suitable thermomechanical properties to formulate sustainable vegetable oil-based drilling fluids. In addition, nanoparticles have been extensively used as stabilizers in drilling fluid emulsions, given their extensive surface/volume ratio, as reported by several authors [21,22,23].

Less attention has been devoted to the use of nanoparticles as rheology improvers for oil suspensions. Fumed silica particles have the capability to change the rheological behavior of vegetable oil suspensions, providing a large variety of non-Newtonian behaviors, from gel-like to shear-thinning, over a wide range of temperatures, depending on the concentration and the nanosilica’s hydrophobicity [24,25,26]. Therefore, a thermomechanical characterization over a wide range of pressures and temperatures are required to assess the conditions under which suspensions of vegetable oils structured with mineral fillers could replace traditional oil-based fluids.

This work is part of a comprehensive study on the development of environmentally-friendly fluids for drilling applications, using nanoparticles as rheology improvers dispersed in a continuous phase of vegetable origin. In a previous work, suspension of organosepiolites in vegetable oil were studied [20]. Herein, both the microstructure and the rheological behavior of hydrophobic fumed silica (HFS) dispersed in a sunflower oil are explored, as a function of temperature and pressure.

## 2. Materials and Methods

### 2.1. Materials

Sunflower crude oil (S, acidity 0.37 wt% oleic acid) was donated by LIPSA (Barcelona, Spain). Fatty acid compositions reported by the supplier appear in Table 1.

Commercial HFS (AEROSIL R106, R805, and R974) were purchased from Evonik (Essen, Germany). Some characteristics of HFS provided by the supplier appear in Table 2.

HFS suspensions were prepared by dispersing a 3 wt% of HFS in crude sunflower oil at room temperature. The dispersion was homogenized for five minutes using a Silverson L5 high shear mixer (Silverson Machines Ltd., Chesham, UK), at a speed of 6000 rpm. The suspensions were referred as: R106S, R805S, and R974S. They were stored in closed containers at 60 °C, for 24 h, to remove air bubbles. Before testing, samples were inspected and homogenized for 5 min in a low shear mixer; high stability was observed during storage.

### 2.2. Thermogravimetric Analysis

Thermogravimetric characterization was performed using a Thermogravimetric Analyzer Q50 (TA Instruments, New Castle, DE, USA). Samples of 5–15 mg were isothermally stabilized at 25 °C for 5 min, heated from 25 to 600 °C, at a heating rate of 10 °C/min, under a N_2_ purge flow of 5 mL/min.

### 2.3. Calorimetric Analysis

Calorimetric characterization was developed using a Modulated Differential Scanning Calorimeter Q100 (TA Instruments, New Castle, DE, USA). Samples of 5–15 mg were sealed in aluminum pans. A cooling/heating rate of 5 °C/min, an oscillation period of 50 s, an amplitude of 0.50 °C and N_2_ purge flow of 50 mL/min were selected for all tests. Suspensions were subjected to the following four cooling/heating cycles: 1st cooling cycle: from 80 to −80 °C, 1st heating cycle: from −80 to 250 °C, 2nd cooling cycle: from 250 to −80 °C, 2nd heating cycle: from −80 to 80 °C. In addition, before each cycle, the samples were isothermally stabilized for 5 min at the starting temperature.

### 2.4. Rheological Characterization

Rheological characterization was carried out with a controlled-stress rheometer Mars II (Thermo Fisher Scientific, Waltham, MA, USA). Viscosity at atmospheric pressure were obtained with a conventional coaxial cylinder geometry Z20DIN. Viscosity-pressure data were obtained using a patented high-pressure flow driven capillary viscometer, equipped with a high-pressure opposed-piston pump, pressure generators and a set of high-pressure capillaries enclosed in a climate chamber (L/D from 12,000 to 3000); details of this device are reported elsewhere [27].

Steady-state flow curves, at 40, 60, 80, 100, 120, and 140 °C, were measured using upward and downward stress sweep steps during a time of 60 s. The experimental error between upward and downward curves was less than 5%.

Step viscosity-pressure ramps, at increments of 200 bar, were measured in the HP capillary viscometer in the range of 1–2500 bar, using high-pressure generators (High Pressure Equipment Co, Erie, PA, USA; Teledyne Isco Inc., Lincoln, NE, USA; Sitec-Sieber Engineering AG, Maur, Switzerland), and the sample as pressurizing fluid.

### 2.5. Aging Procedure

Dynamic aging was carried out in the pressure cell D400/200 (diameter D = 39 mm and height H = 140 mm). A 110 mL volume of the sample was poured into the pressure cell and was pressurized with N_2_ at initial ΔP ≈ 40 bar at 40 °C. After 30 min of stabilization, it was heated from 40 to 190 °C, by 1 °C·min^−1^, and was then aged at 190 °C for 20 h, to finally cool down by 1 °C·min^−1^ to room temperature. During the aging process, viscosity was measured with non-conventional geometries (double helical ribbon of 36 mm diameter (DHR-2) and 78 mm length impeller (FL4)) at the controlled rate of 200 rpm. The rate was selected to reach limiting viscosity. Non-conventional geometries were previously calibrated using the procedure described by Hermoso et al. [28].

## 3. Results and Discussion

### 3.1. Thermal Characterization

**Figure 1:**
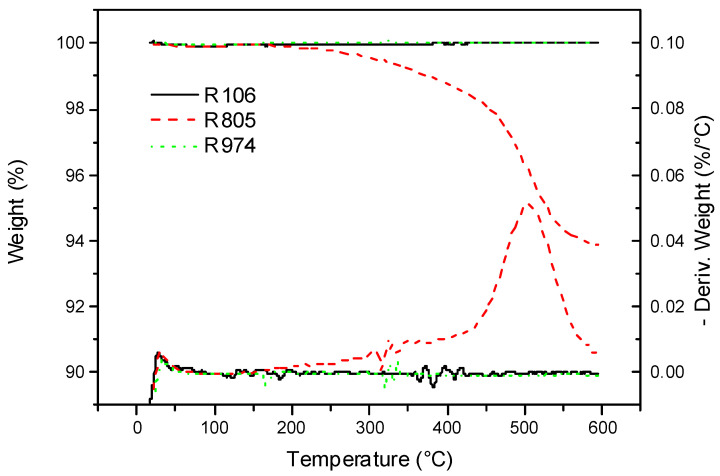
TGA thermograms for studied HFS samples at a heating rate of 10 °C·min^−1^.

As Figure 1 shows, HFS samples are very stable regarding temperature. These HFS samples had a weight loss of less than 0.05% from room temperature to 200 °C, probably caused by an insignificant surface water loss. Samples R106 and R974 revealed the highest thermal stability, retaining their weight in the whole range of temperatures tested (25–590 °C). This indicates that bound Si-CH3 is highly resistant to temperature in this temperature range [29] and no significant decomposition of the hydrophobic groups is observed by TGA (see Table 3).

On the other hand, the R805 sample lost 1 and 5% of its weight at 376.1 and 528.5 °C due to decomposition of the aliphatic tail, with an onset at 444.5 °C and a peak at 503.0 °C, with a weight loss of 6.13% at 590 °C, temperature at which the complete degradation of the aliphatic chain took place. Nevertheless, this sample reveals suitable thermal resistance for use as a component in OBM formulations.

**Figure 2:**
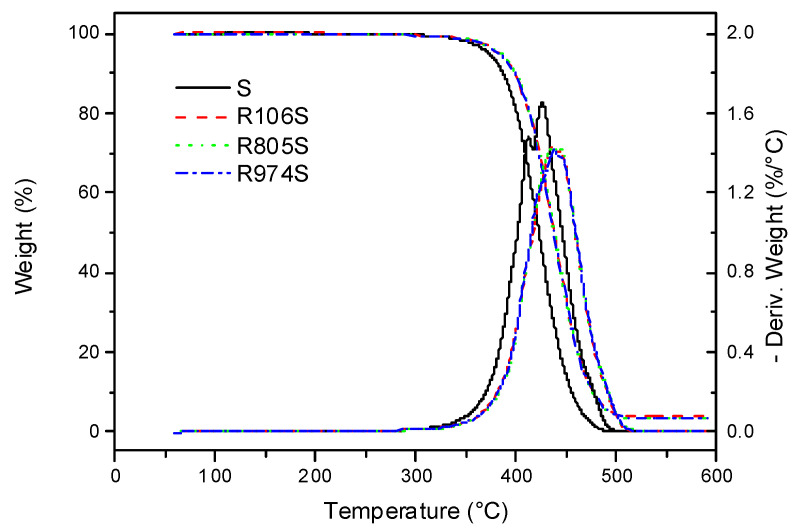
TGA thermograms of base oil and HFS suspensions at a heating rate of 10 °C·min^−1^.

Figure 2 displays the thermogram of the base oil and vegetable oil suspensions formulated with a 3 wt% of the different HFS. As Figure 2 shows, both the vegetable oil and the HFS suspensions show high thermal resistance. The sunflower oil sample had a 1% weight loss at 330.8 °C. Similar resistance was previously reported for this vegetable oil [30]. Suspensions had a weight loss of 1% at similar or higher temperatures than those of the base oil, with the R805S suspension having the highest thermal stability and R974S, the lowest. This may be deduced from the decomposition temperature values shown in Table 3. The weight loss values for the suspensions were corrected according to Tarrío-Saavedra et al. [31], considering both the residue at 590 °C and the weight loss of pure HFS displayed in Figure 1. These results indicate the positive effect exerted by the interactions between HFS and oil on the oil’s decomposition processes, shifting the 1% weight loss to slightly higher temperatures in the case of R106S and R805S suspensions.

A 5% weight loss takes place at 372.4 °C for the oil and 10 °C higher, around 381 °C, for the suspensions, indicating that very similar decomposition mechanisms occur for all HFS samples. In this region, the HFS-oil interaction also has a positive effect on the suspension’s decomposition processes. These phenomena are more significant for the suspension formulated with R805, which once again reveals the highest decomposition temperature at 383.8 °C, even though the weight loss from decomposition of HFS R805 approaches 1% at this temperature (see Figure 1). Finally, onset and maximum temperatures reveal the same tendency as characteristic weight loss, as seen in Table 3. These results reveal that HFS suspensions show more resistance against thermal decomposition than the base oil, especially the R805S suspension.

**Figure 3:**
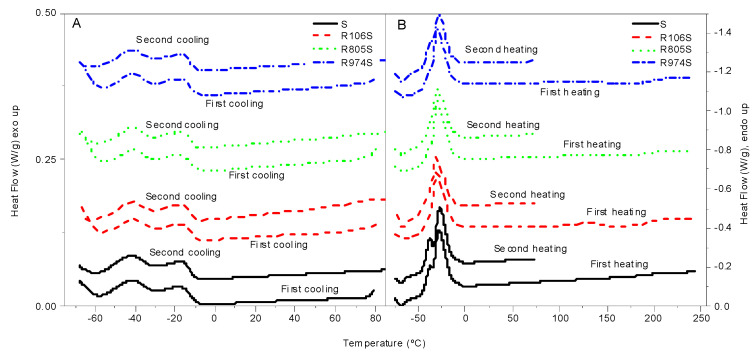
MDSC thermograms for HFS suspensions studied. (**A**) Cooling curves. (**B**) Heating curves.

As can be seen in Figure 3, crude sunflower oil exhibits a wide crystallization region in the first cooling from ambient temperature (Figure 3A). Three characteristics events appear: A onset, at −9.1 °C, the cloud point related to the incipient crystallization of higher molecular weight components; an initial crystallization peak (pk1), at −17.5 °C, related to the formation of microcrystal; and a second peak (pk2), at −41.2 °C, due to the arrangements and recrystallizations of microcrystals to form polymorphic structures [32,33,34].

The first heating curve, up to 250 °C (Figure 3B), shows a broad exothermic melting event at −27.6 °C, with a small peak at −37.6 °C, related to the melting of low molecular weight components. The second cooling, from 250 °C, displays the same thermal events as those of the first one, with a minor shifting to lower temperatures (0.5 °C), probably due to a more effective solution of the residual waxes that could remain at ambient temperature. A similar tendency is observed in the second heating, which shows the same event as the first heating scan, with shifting to higher temperatures, as seen in Table 4.

Minimal difference in the crystallization events were obtained comparing the cooling curves of the oil and the HFS suspensions. Formulations containing different types of HFS do not exhibit distinct thermal event temperatures or crystallization curve shapes. In the case of melting curves, a unique peak appears with the maximum being slightly shifted to lower temperatures. Consequently, HFS does not act as a nucleating agent for oil crystallization. However, the presence of HFS joins the melting process into one unique event, without the separate melting of the low molecular fraction of the oil.

### 3.2. Rheological Characterization

**Figure 4:**
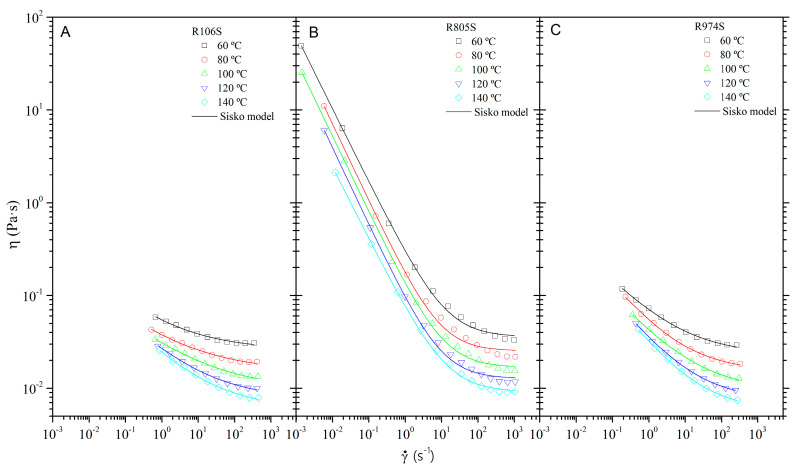
Flow curves of HFS suspensions as a function of shear-rate at different temperatures. (**A**) Suspension R106S. (**B**) Suspension R805S. (**C**) Suspension R974S. (Solid lines represent the Sisko model).

Figure 4 shows the viscosity curves at atmospheric pressure for the HFS suspensions in the range of temperature 60–140 °C. Pseudoplastic behavior was observed in the whole range of temperature and shear rate tested. Interactions between the HFS particles dispersed in oil phase develops a flocculated structure formed by interconnected particle aggregates [35]. The colloidal forces would be responsible for the increasing viscosity values. This behavior is characteristic of fumed silica in oil suspensions above a threshold concentration, approximately 3–4 wt%, as previously reported by Khan and Zoeller [36]. As the shear-rate increases, the structure of the interconnected associations progressively vanishes. The breakdown of the aggregate’s assembly and the orientation under flow decrease the interactions in the dispersed phase and, therefore, the viscosity of the suspension [37].

In the high shear-rates region, the suspension trends to a limiting value of the viscosity higher than that of the Newtonian oil [38]. In this region, the suspension’s viscosity depends mainly on the hydrodynamic volume fraction of the particles and aggregates. The maximum orientation under flow, the minimum interactions between particles and aggregates in this region.

The viscous flow behavior of fumed silica suspensions can be modeled by the Sisko model:

(1) $$\eta =\eta_{\infty }+k_{0}\cdot {\dot{\gamma }}^{n_{0}-1}$$

where $\dot{\gamma }$ is the shear-rate, *k*_0_ is the consistency index, *n*_0_ is the flow index, and $\eta_{\infty }$ is the high-shear-rate-limiting viscosity. As Figure 4 displays, the Sisko model adequately describes the flow curves of the HFS suspensions. Higher values of the viscosity are reached at lower temperatures. The increase in temperature conduct to a decrease in the limiting viscosity values, which depends on the nature of the HFS used to prepare the suspension.

As seen in Table 5, despite the different average primary particle size of R106 and R974 (see Table 2), the suspensions prepared with R106 and R974 silica have limiting viscosity values that are quite similar to one another and are higher than those of the oil at each temperature. The differences in limiting viscosity values disappear as the temperature increases, indicating the influence of Brownian motion on the degree of structuring of HFS in the system. The increase in viscosity is likely to be more closely related to the association of the HFS particles than to the individual size of the particle. These associations would form agglomerates that are highly sensitive to temperature. In contrast, the suspension prepared with R805 has the highest increase in limiting viscosity with respect to oil. This increase is practically independent of temperature, having twice the viscosity of the base oil in all temperature ranges. In addition, this suspension also has the highest values of the consistency index and the lowest values of the flow index, indicating that the presence of the octyl tail (see Table 2) leads to a higher degree of suspension structuring, improving the pseudoplastic properties.

As observed, the consistency index of the Sisko model (Equation (1)) decreases with temperature for all suspensions, probably the increase of Brownian motion decreases the degree of flocculation of the system. Nevertheless, the flow index remains practically constant, with random oscillations in the temperature range tested.

Shear-thinning behavior is required for drilling fluids. A low viscosity saves energy at higher circulation rates. A high viscosity avoids the settling process of the solids when the circulation is stopped. In this sense, not all HFS suspensions in vegetable oil studied have the required properties as rheology modifiers for drilling fluids. The degree of pseudoplasticity of R106S and R974S suspensions appears to be lower than that of organoclay suspensions in the same concentration range [11,12,20]. Nevertheless, the HFS suspension formulated with R805 reveals pseudoplastic properties that cover the requirement of high viscosity at low shear-rate region and low viscosity at high shear-rate.

### 3.3. Thermal Aging

**Figure 5:**
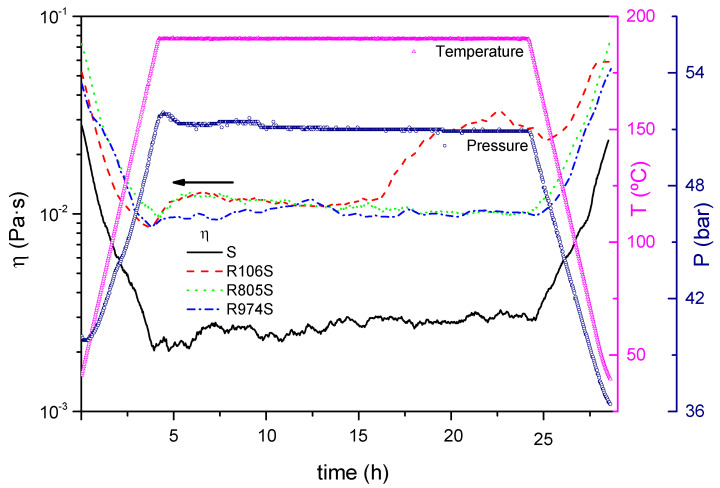
Evolution of viscosity with aging time for the HFS suspensions studied.

Figure 5 shows the evolution of the viscosity of the suspensions with the thermal aging time at 190 °C. The viscosity of the oil increases slightly with the aging time as a result of the incipient degradation processes. Nevertheless, all the components of the suspension show excellent thermal stability at temperatures above 200 °C, as corroborated by the TGA (Figure 1 and Figure 2).

A good stability measured by the evolution of the viscosity during aging is observed for all suspensions. The suspension formulated with R106 showed an increase of approximately twice the initial viscosity after 12 h of aging time.

**Figure 6:**
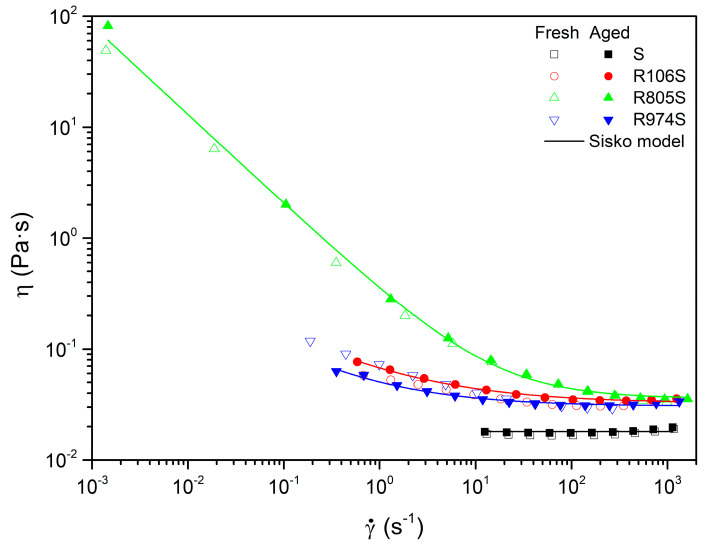
Effect of aging on the pseudoplasticity of HFS suspensions at 60 °C (solid lines represent the Sisko model).

Aged suspensions have been characterized at a control temperature selected at 60 °C. A comparison of the flow curves for fresh and aged suspensions is displayed in Figure 6. It may be observed that a good agreement exists between the fresh and aged curves. It has been reported that fluids based in vegetable oil may suffer hydrolysis degradation at high temperatures which would increase viscosity [2]. In this case, all suspensions maintained similar flow behavior before and after aging, showing a great stability against degradation at high temperatures. Consequently, the effect of aging on the parameters of the Sisko model was quite minimal. Suspension R805S appears to be the most stable in terms of aging. Suspensions R106S and R975S show an increase in the limiting viscosity until the value of R805S, and a slight decrease in flow indexes, as deduced by comparing the values of Table 5 and Table 6.

### 3.4. Pressure Behavior

**Figure 7:**
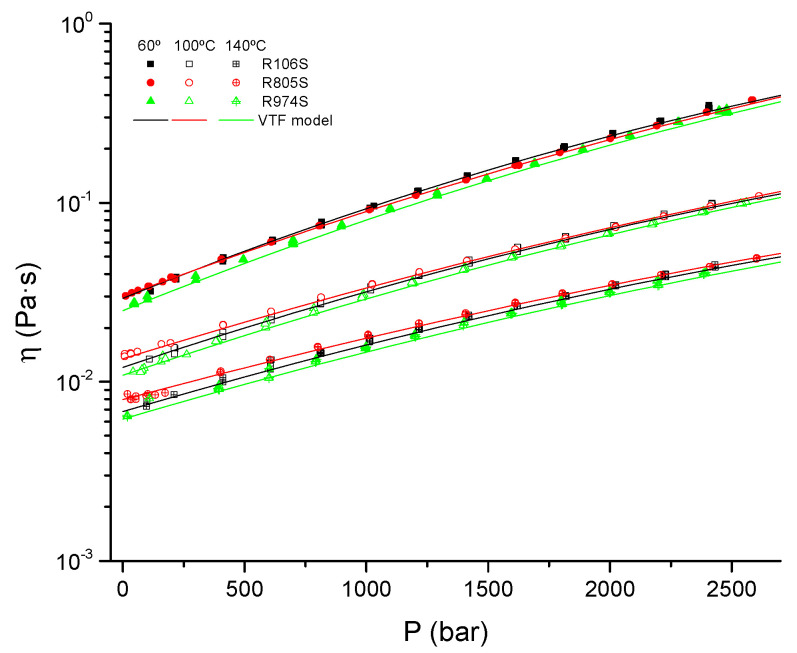
Pressure-viscosity curves for fumed silica suspensions for selected temperatures.

The rheology of OBM is more sensitive to pressure than that of WBM due to the low molecular weight of the organic components in the continuous phase [39,40]. In this sense, to model the viscosity-pressure behavior at high circulation rates, viscosity versus pressure at a constant temperature, were measured in both rotational and capillary flow, at a shear-rate in the range of 10^3^–10^4^ s^−1^. In this range, the viscosity of all suspensions approaches the high-shear-rate-limiting value. The results of the pressure-viscosity tests at selected temperatures are presented in Figure 7.

At constant temperature, viscosity-pressure curves reveal a progressive deviation from linearity in log scale, mainly in the high-pressure region. Therefore, the use of simple exponential models, such as the Barus model, to assess the HPHT behavior would result in an overestimation of the effect of pressure. These results corroborate the limitations of the simple exponential pressure models to cover wide ranges of pressures. Consequently, more complex equations have been proposed [41,42]. The combined effect of pressure and temperature on OBM can be satisfactorily modeled using factorial models such as the VTF model [43].

(2) $$\eta =A\cdot exp\left(a_{1}\left(P-P_{r}\right)+a_{2}{\left(P-P_{r}\right)}^{2}+\frac{\left(B+b_{1}\left(P-P_{r}\right)+b_{2}{\left(P-P_{r}\right)}^{2}+b_{3}{\left(P-P_{r}\right)}^{3}\right)}{T-C}\right)$$

where *T* is the temperature, *P* is the pressure, *P_r_* is the reference pressure, *A*, *B*, and *C* are parameters of Vogel’s model and *a*_1_, *a*_2_, *b*_1_, *b*_2_, and *b*_3_ are empirical parameters that quantifies the effect of pressure. The values of the parameter for the studied samples are shown in Table 7.

The VFT equation has been used to generalize the viscosity-pressure-temperature behavior of fluid based on vegetable oils across a wide range of temperatures and pressures [20,43].

**Figure 8:**
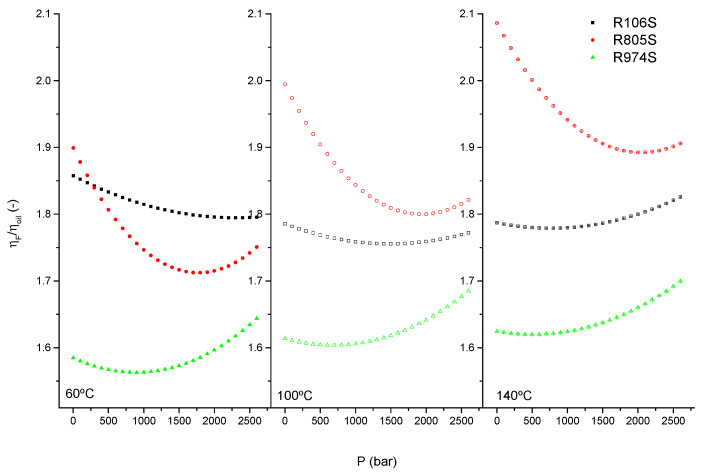
Evolution of the relative viscosity versus pressure for selected temperatures, 60, 100 and 120 °C.

To analyze the combined effect of both pressure and temperature on viscosity, Figure 8 displays the evolution of the relative viscosity (fluid/oil viscosity-pressure ratio), at selected temperatures for the studied suspensions.

HFS suspensions demonstrate a significant increase in viscosity as compared to the base oil. This increase depends on both the pressure-temperature range and the degree of hydrophobicity of silica. At lower temperature-pressures, the lower the carbon content and hydrophobicity in silica (see Table 2), the lower the increase in relative viscosity observed. Relative viscosity for all suspensions exhibits a minimum value at intermediate pressures. As the pressure increases, the relative viscosity increases more significantly at higher temperatures. Therefore, the suspension’s thermal susceptibility with respect to the vegetable oil is reduced in the HPHT region. Suspension R974S and R106S are less sensitive to pressure-temperature than suspension R805S. Therefore, R805S shows both the highest relative viscosity values and the highest decrease with pressure, more evident under HPHT conditions.

This behavior may be explained by the effects of temperature and pressure on both the free volume and the degree of silica agglomeration. Temperature increases the effective volume fraction and decreases the degree of agglomeration. Therefore, as the viscosity of the oily continuous phase decreases, the relative contribution to the bulk viscosity of the dispersed phase (interaction among aggregates) increases, despite the deflocculation of agglomerates, resulting in an increase in the fluid’s relative viscosity [44].

Pressure reduces the effective volume fraction, decreasing the relative contribution of the dispersed phase to the bulk properties of the suspension. Therefore, the relative viscosity decreases are mainly determined by the continuous phase and are more significant in suspensions in which the contribution of the flocculation of silica particle agglomerates is significant, such as R805S. At higher pressures, however, the increase in flocculation of aggregates would compensate for the contribution of the continuous phase, flattening the decrease in relative viscosity or even dominating the increasing values of relative viscosity in the HPHT region. Nevertheless, a precise measurement of the evolution of both expansivity and compressibility of the free volume [41] as a function of temperature and pressure should be performed to evaluate this effect.

## 4. Conclusions

HFS in vegetable oil suspensions reveals adequate thermal resistance to be used as a component in sustainable drilling formulations. The low temperature limit applied to drilling fluids is determined by the crystallization characteristic of the base oil since fumed silica does not act as a nucleating agent for oil crystallization. Nevertheless, silica enhances the resistance to thermal decomposition of the base oil, with the high temperature limits of application being determined by the aging resistance.

HFS suspensions exhibit a significant increase in viscosity as compared to the sunflower oil. The lower the carbon content and hydrophobicity of silica, the lower the increase in relative viscosity observed. Relative viscosity of the oil presents a minimum value at intermediate pressures and increases at high pressure and temperatures, reducing the oil’s thermal susceptibility.

The fluid formulated with sunflower oil and HFS R805 shows a significant improvement in pseudoplastic properties due to the presence of the octyl tail, which enhances the degree of structuring, offers good resistance and stability to thermal aging, and reduces the effect of pressure, being suitable for sustainable oil-based fluids at a wide range of temperatures, pressures, and shear rates.

## Figures and Tables

**Table 1:** Sunflower oil fatty acid content.

C12:0 Lauric (wt%)	C14:0 Myristic (wt%)	C16:0 Palmitic (wt%)	C18:0 Stearic (wt%)	C18:1 Oleic (wt%)	C18:2 Linoleic (wt%)	C18:3 Linolenic (wt%)	Others (wt%)
0.00	0.06	6.15	3.30	24.83	64.48	0.06	1.12

**Table 2:** Physicochemical characteristics of the HFS.

	BET (m^2^/g)	Average Primary Particle Size (nm)	C Content (wt.%)	Fumed Silica Modified with:
R106	250 ± 30	7	1.4–3.0	Octamethylcyclotetrasiloxane 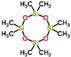
R805	150 ± 25	12	4.5–6.5	Trimethoxy(octyl)silane 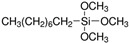
R974	170 ± 20	12	0.7–1.5	Dimethyldichlorosilane 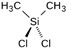

**Table 3:** Characteristic values of weight loss from TGA for the studied samples.

	Degradation Temperatures (°C)			
Samples	T_1_ (°C) (T at 1% Weight Loss)	T_5_ (°C) (T at 5% Weight Loss)	T_onset_ (°C)	T_peak_ (°C)
S	330.8	372.4	391.0	426.0
R106S	334.0	381.8	402.1	434.2
R805S	337.0	383.8	402.3	437.3
R974S	330.9	391.9	401.7	437.6

**Table 4:** MDSC thermal events from the cooling and heating cycles of the samples studied.

Sample	Crystallization (°C)			Melting (°C)		
	Onset (°C)	Peak 1 (°C)	Peak 2 (°C)	Peak 1 (°C)	Peak 2 (°C)	ΔH_m_ (J/g)
S-1st	−9.1	−17.5	−41.2	−37.5	−27.9	74.6
S-2nd	−9.8	−18.5	−41.7	−37.8	−27.4	
R106S-1st	−8.7	−18.9	−40.9		−30.1	54.8
R805S-2nd	−9.0	−18.3	−41.2	-	−31.2	
R805S-1st	−9.4	−18.1	−41.3	-	−29.6	46.6
R106S-2nd	−8.9	−18.7	−41.0	-	−29.5	
R974S-1st	−9.2	−18.8	−40.9	-	−29.7	56.6
R974S-2nd	−9.7	−17.7	−41.2	-	−28.3	

**Table 5:** Parameters of the Sisko model for the HFS suspensions studied.

	Limiting Viscosity (*η*_ꝏ_), Pa·s					Consistency Index (*k*_0_), Pa·s^n^					Flow Index (*n*_0_)				
Samples	60 °C	80 °C	100 °C	120 °C	140 °C	60 °C	80 °C	100 °C	120 °C	140 °C	60 °C	80 °C	100 °C	120 °C	140 °C
S	17.08 × 10^−3^	10.01 × 10^−3^	7.09 × 10^−3^	5.16 × 10^−3^	3.83 × 10^−3^										
R106S	26.18 × 10^−3^	15.33 × 10^−3^	9.79 × 10^−3^	7.75 × 10^−3^	5.95 × 10^−3^	0.028	0.022	0.021	0.019	0.018	0.638	0.671	0.672	0.614	0.599
R805S	35.91 × 10^−3^	25.12 × 10^−3^	16.76 × 10^−3^	12.72 × 10^−3^	9.18 × 10^−3^	0.269	0.153	0.121	0.085	0.068	0.208	0.168	0.181	0.167	0.223
R974S	23.43 × 10^−3^	15.45 × 10^−3^	9.65 × 10^−3^	7.59 × 10^−3^	5.82 × 10^−3^	0.047	0.04	0.033	0.028	0.026	0.577	0.517	0.557	0.509	0.492

**Table 6:** Effect of aging on the parameters of the Sisko model.

Aged Suspensions	Limiting Viscosity (*η*_ꝏ_), Pa·s	Consistency Index (*k*_0_), Pa·s^n^	Flow Index (*n*_0_)
Aged_S	17.99 × 10^−3^	-	-
Aged_R106S	32.53 × 10^−3^	0.032	0.515
Aged_R805S	35.85 × 10^−3^	0.322	0.197
Aged_R974S	30.54 × 10^−3^	0.020	0.456

**Table 7:** Parameters of the VTF model for the analyzed samples.

	S	R106S	R805S	R974S
*A*/Pa·s	2.13 × 10^−4^	3.60 × 10^−4^	6.75 × 10^−4^	3.35 × 10^−4^
*B*/°C	698.1	733.3	565.5	720.6
*C*/°C	−102.3	−104.3	−89.37	−107.2
*a*_1_/bar^−1^	2.23 × 10^−4^	8.72 × 10^−5^	2.24 × 10^−4^	2.32 × 10^−4^
*a*_2_/bar^−2^	0	0	0	0
*b*_1_/°C·bar^−1^	0.1740	0.1778	0.1424	0.1726
*b*_2_/°C·bar^−2^	−1.90 × 10^−5^	−1.35 × 10^−5^	−1.26 × 10^−5^	−1.67 × 10^−5^
*b*_3_/°C·bar^−3^	0	0	0	0
*P_r_*/bar	1	1	1	1

## Data Availability

The raw/processed data required to reproduce these findings cannot be shared at this time due to technical or time limitations.
